# Psychological determinants of the effectiveness of conjugated linoleic acid supplementation in overweight and obese women—a randomized controlled trial

**DOI:** 10.3389/fnut.2024.1342452

**Published:** 2024-07-19

**Authors:** Małgorzata Jamka, Joanna Popek, Anna Bukowska-Posadzy, Edyta Mądry, Aleksandra Lisowska, Katarzyna Jończyk-Potoczna, Judyta Cielecka-Piontek, Paweł Bogdański, Jarosław Walkowiak

**Affiliations:** ^1^Department of Pediatric Gastroenterology and Metabolic Diseases, Poznan University of Medical Sciences, Poznan, Poland; ^2^Department of Clinical Psychology, Poznan University of Medical Sciences, Poznan, Poland; ^3^Department of Physiology, Poznan University of Medical Sciences, Poznan, Poland; ^4^Department of Pediatric Diabetes, Auxology and Obesity, Poznan University of Medical Sciences, Poznan, Poland; ^5^Department of Pediatric Radiology, Poznan University of Medical Sciences, Poznan, Poland; ^6^Department of Pharmacognosy and Biomaterials, Poznan University of Medical Sciences, Poznan, Poland; ^7^Department of Treatment of Obesity, Metabolic Disorders and Clinical Dietetics, Poznan University of Medical Sciences, Poznan, Poland

**Keywords:** healthy behaviors, psychological factors, wellbeing, self-esteem, self-efficacy

## Abstract

**Introduction:**

Previous studies investigating the effectiveness of conjugated linoleic acid (CLA) supplementation in body weight reduction provided unequivocal results. We hypothesized that psychological factors such as self-efficacy, locus of control or dispositional optimism can affect the success of the intervention. Therefore, this study aimed to determine the psychological factors that modulate the effectiveness of CLA supplementation in overweight or obese women and affect the ability to successfully complete the study.

**Methods:**

In total, 74 subjects were recruited into this three-month randomized trial and divided into intervention and control groups receiving, respectively, capsules containing 3 g 80% CLA per day and capsules containing 3 g of sunflower oil. The following psychological tests were performed before the intervention: Multidimensional Health Locus of Control scale, Acceptance of Illness Scale, Satisfaction with Life Scale (SWLS), General Self Efficacy Scale (GSES), Health Behavior Inventory scale and Life Orientation Test (LOT-R).

**Results:**

A total of 60 women completed the study and the subjects who dropped out obtained higher scores in the GSES (*p* = 0.0490) and the LOT-R (*p* = 0.0087) tests than subjects who completed the trial. Besides, multivariate linear regression demonstrated that the SWLS test (*p* = 0.0345) results were independent predictors of body weight changes.

**Conclusion:**

In conclusion, psychological factors like self-efficacy and optimism may be associated with a higher risk of withdrawal from the study, while satisfaction with life may have an impact on the effectiveness of body weight reduction.

**Clinical trial registration**: [https://drks.de/search/en], identifier [DRKS00010462].

## 1 Introduction

Obesity is considered a serious threat to public health worldwide, the prevalence of which has dramatically increased among both men and women in all age groups. Since 1980, the excessive body weight rate has doubled, with around a third of the global population being overweight or obese (1). Moreover, it is expected that 1.9 billion people worldwide will be living with obesity by 2035 (2). Several weight management strategies have been suggested, including diet restriction, physical activity, behavioral strategies, pharmacology and bariatric surgery (3). Weight loss is associated with numerous health and psychological benefits, including decreased risk of type 2 diabetes mellitus, cardiovascular diseases, chronic kidney disease, sleep apnea or asthma (4). Despite these benefits, body weight reduction is often difficult to achieve. A meta-analysis encompassing 29 long-term weight loss studies revealed that over half of the lost weight was regained within 2 years, and by the end of 5 years, more than 80% of the lost weight was recovered (5). Therefore, the identification of psychological factors that may determine the success of different strategies for body weight reduction is particularly important. The American Dietetic Association recommends that psychological factors should be assessed together with dietary intake to optimize subjects’ response to body weight loss interventions and to maintain body weight reduction (6). However, to date, the search for psychological factors (e.g., self-efficacy, locus of control, dispositional optimism) that determine the effectiveness of different strategies in body weight reduction has yielded inconsistent findings (7–13).

One of the dietary supplements with potential body weight reduction properties is conjugated linoleic acid (CLA). CLA is a comprehensive term used to describe isomers of octadecadienoic (linoleic) acid that contain conjugated double bonds (14). Ruminants possess an enzyme capable of converting fatty acids into CLA. Thus, food from ruminant sources (meat and dairy products) is a natural source of CLA (15). CLA can also be synthesized using oils abundant in linoleic acid, including safflower and sunflower oils (16). It has been shown that CLAs have various beneficial effects on atherosclerosis, cancer (17), diabetes (18), and inflammatory process (19). However, the anti-obesity effect of CLA is controversial. Onakpoya et al. (20) in the meta-analysis showed that long-term CLA supplementation decreased body weight, body mass index (BMI) and fat mass when compared to placebo. However, the effect was small and did not seem clinically relevant. Contrary, Liang et al. (21) in a recent meta-analysis demonstrated that CLA supplementation during exercise programs was not effective for body-weight control compared to exercise alone but significantly reduced fat mass.

Taking into account the inconsistency in the obtained results we hypothesized that psychological factors might affect the success of the CLA supplementation. Therefore, this study aimed to determine the psychological factors that modulate the effectiveness of CLA supplementation in body weight reduction in overweight or obese women and affect the ability to successfully complete the study.

## 2 Materials and methods

### 2.1 Study design

The study was a parallel randomized controlled trial and was performed between July 2014 and May 2015. The Poznan University of Medical Sciences Ethics Committee approved the study protocol (ref. 606/12, 453/13, 358/14, and 398/15) and the research was performed according to the Declaration of Helsinki (22). All study participants were informed that participation was voluntary and that they can withdraw from the study at any time without giving a reason. Informed consent was obtained from all individual participants included in the study. This paper was written according to the consolidated standards of reporting trials (CONSORT, see Supplementary Table S1) (23) and the study project was retrospectively registered in the German Clinical Trials Register database (ID: DRKS00010462, date of registration: 04.05.2016) (24).

### 2.2 Inclusion and exclusion criteria

The inclusion and exclusion criteria were as described previously (25–30). Briefly, women older than 18 years old with a BMI ≥ 25 kg/m^2^ and stable body weight (± 3 kg during the last 3 months) were recruited. Subjects with a history of chronic diseases, including celiac disease, type 2 diabetes mellitus, and hepatic and pancreatic diseases, were excluded. Pregnant and breastfeeding women and subjects who previously received CLA supplementation or taking dietary supplements which interfered with fat digestion or absorption were also excluded. Study screening was performed at the Department of Internal Medicine, Metabolic Disorders and Hypertension, Poznan University of Medical Sciences, Poland.

### 2.3 Interventions protocol

During the 12-week intervention period, the CLA group received six capsules per day containing 3 g 80% CLA (0.5 g per capsule), a 50:50 mixture of cis-9, trans-11 and trans-10, and cis-12 isomers. The placebo group received six identical capsules per day, each containing sunflower oil. The capsules were manufactured and packaged in blisters by the Olimp Laboratories company (Pustynia, Poland). Compliance was monitored by phone calls, with the women consuming at least 75% of the capsules included in the analysis. Participants were instructed not to change their dietary habits and physical activity during the intervention.

### 2.4 Outcomes

The main outcomes of the study included an assessment of psychological behaviors that could determine the effectiveness of the study intervention. However, this manuscript is part of a larger project which aimed to evaluate the effect of CLA supplementation on starch and lipid digestion using a breath test (29) and the secondary purpose of the project was to assess the effect of CLA supplementation on anthropometric parameters (25), body composition and liver markers (26), atherosclerosis parameters (28), densitometric variables (30), glucose and insulin homeostasis, lipid metabolism and adipokine levels, as reported previously. All outcomes were assessed at the Department of Pediatric Gastroenterology and Metabolic Diseases, Poznan University of Medical Sciences, Poland.

### 2.5 Anthropometric parameters

Anthropometric parameters were measured in the morning with the participants wearing light clothing and without shoes. Body weight and height were measured using the Radwag scale with a stadiometer (Radwag, Random, Poland). BMI was calculated and overweight was defined as BMI ≥ 25 kg/m^2^ and obesity as BMI ≥ 30 kg/m^2^ (1). Subjects who decreased body weight after the intervention period were compared to subjects who increased body weight after the intervention separately in the CLA and control groups.

### 2.6 Psychological tests

The following tests were used: Multidimensional Health Locus of Control scale (MHLC), Acceptance of Illness Scale (AIS), Satisfaction with Life Scale (SWLS), General Self Efficacy Scale (GSES), Health Behavior Inventory scale (IZZ), and Life Orientation Test (LOT-R).

The MHLC includes 18 statements, each assessed on a six-level scale related to beliefs on expectations in three dimensions of the health locus of control: internality (I)—the internal locus of health control, the influence of others (O)—the external locus of health control and random (R)—chance or random locus of health control. Each index ranges from 0 to 30 points, with higher scores indicating a stronger belief in the influence of a particular factor on health. The MHLC scale has two versions: A and B. In this study, we used version A. The reliability of this Polish version of the MHLC scale determined by the Cronbach α was 0.77 (I), 0.67 (O), and 0.76 (R).

The AIS scale comprises eight statements addressing various aspects such as the constraints brought about by the illness, diminished independence resulting from it, the feeling of being dependent on others, and reduced self-esteem. Participants were asked to express their agreement or disagreement with these statements using a five-point scale, where 1—signifies strongly agree; 2—agree; 3—undecided; 4— disagree; 5—strongly disagree. The total score falls within the range of 8–40, with a higher score indicating a greater degree of acceptance of illness. The Polish version of the AIS scale has good psychometric properties, with reliability measured by the Cronbach’s *α* = 0.85.

The SWLS was developed to assess life satisfaction and consists of five items rated on a seven-point Likert scale, where 1 means strongly disagree and 7 means strongly agree. The total score for the scale ranges from 5 to 35, with a lower score indicating a lower level of satisfaction with life, while a high score indicates a higher level of satisfaction with life. The reliability coefficient for the scale is good (the Cronbach’s *α* = 0.88).

The GSES scale is a 10-item self-report scale that measures general self-efficacy. Respondents rate each item on a scale from 1 (not at all true) to 4 (completely true). The total score on the GSES scale can vary between 10 and 40, where higher scores reflect greater levels of self-efficacy. The Cronbach’s α coefficient for the scale is 0.81.

The IZZ scale contains 24 statements describing various health-related behaviors, each assesses on a five-point scale, from “hardly ever,” to “nearly always.” The overall score was computed as the sum of all these statements, resulting in a score range from 24 to 120 points. Moreover, the intensity of four categories of health behaviors was evaluated: eating habits, prophylactic behaviors, a positive mental attitude and health practices. The higher the score, the greater the declared health behavior intensity. The reliability for the total IZZ scale based on the Cronbach’s α coefficient is 0.85.

The LOT-R scale contains 10 statements, with six of a diagnostic value for dispositional optimism and four that were not taken into account in the calculation of the results. A five-point scale was used to evaluate each statement, where 0 points mean that the statement definitely does not apply to me, while 4 points mean that the information definitely applies to me. The overall score is the sum of each statement, including three positive and three negative, so the number of points for the negative statements is inverted before being summed. The overall score ranged from 0 to 24 points with a higher score meaning a higher level of optimism. The Cronbach’s α for the Polish version is 0.76 (31).

### 2.7 Randomization and blinding

Blocking randomization was performed and a randomization list was generated. The allocation sequence remained undisclosed until participants were registered and subsequently allocated to their respective interventions. During the intervention, both study participants and researchers were not aware of the allocation. After the statistical analysis of the obtained results was completed, the study was unblinded and participants were informed about their study group assignment.

### 2.8 Minimum sample size

The Statistica 12 PL software (TIBCO Software Inc., Palo Alto, United States) was used to calculate the minimum sample size indicating that 74 subjects should be recruited to the study to obtain 80% power (*α* = 0.05, *β* = 0.2). Based on the results of our previous studies, we hypothesized that the mean differences in primary outcomes (starch and lipid digestion assessed by a breath test) would explain approximately 75% of the variances that we previously observed (32, 33). In addition, in the calculation, we assumed that a maximum of 20% of participants resigned from the study during the intervention.

### 2.9 Statistical analysis

The Statistica 13 PL software (TIBCO Software Inc., Palo Alto, United States) was used for statistical analysis and *p* < 0.05 was considered statistically significant. Mean, standard deviation (SD), 95% confidence interval (95% CI), median and interquartile range (IQR; Q1–Q3) were calculated. The Shapiro–Wilk was used to assess the normality of the distribution of variables. The Mann–Whitney U-test was used to examine differences between the study groups (CLA group vs. control group), individuals who completed and those who did not complete the study and subjects who decreased and increased body weight after the intervention period. Univariate and multivariate linear regression analysis was performed to assess the relationship between changes in body weight and psychological test results.

## 3 Results

### 3.1 Participants’ flow

**Figure 1:**
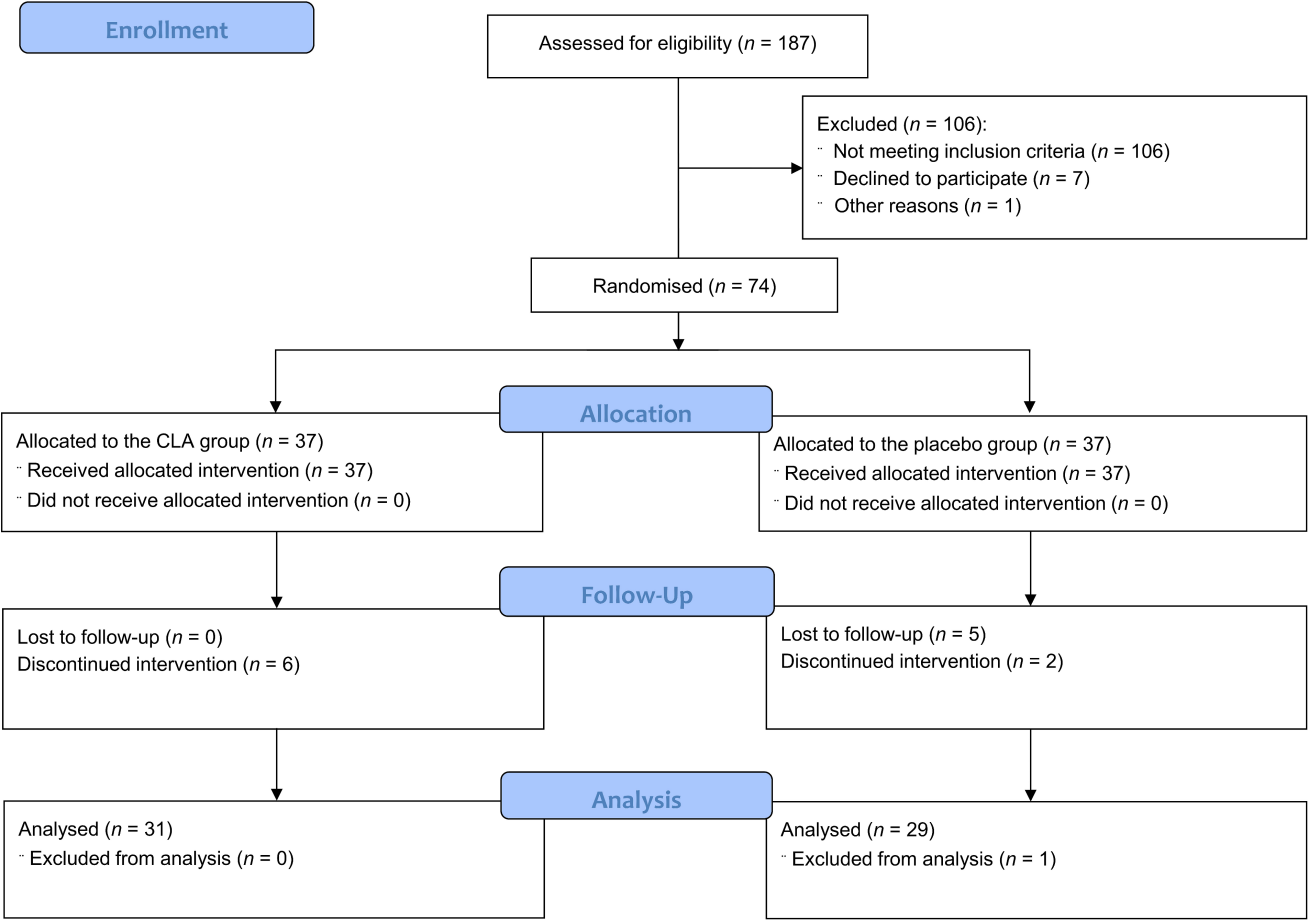
CONSORT 2010 participants flow diagram.

The study workflow is presented in Figure 1. In total, 187 subjects were assessed to meet inclusion and exclusion criteria in the Department of Internal Medicine, Metabolic Disorders and Hypertension, Poznan University of Medical Sciences, Poland. Among them, 81 women were selected for the study. Seven participants refused to participate, so 74 individuals were randomly divided into two groups: the CLA group (*n* = 37) and the placebo group (*n* = 37). Moreover, one woman refused to complete the psychological tests and was excluded from the analysis. Sixty participants completed the intervention and were included in the final analysis, with no serious adverse effects reported. Two participants reported nausea and one woman had a rash. The baseline characteristics of the study population were presented previously (25, 26, 29). There were no differences between the CLA and control groups in the results of psychological tests for subjects who were included in the study (Table 1). Similarly, when we compared subjects who completed the study, we noted no significant differences between groups (Table 2).

**Table 1:** Comparison of the results of psychological tests between subjects who were included to the study.

		Total (*n* = 73)		CLA group (*n* = 37)		Control group (*n* = 36)		*p*
		Mean ± SD (95%CI)	Median (IQR)	Mean ± SD (95%CI)	Median (IQR)	Mean ± SD (95%CI)	Median (IQR)	
MHLC	I	27.48 ± 4.79 (26.36; 28.60)	28.00 (25.00; 30.00)	28.14 ± 5.06 (26.45; 29.82)	29.00 (25.00; 31.00)	26.81 ± 4.46 (25.29; 28.32)	27.00 (24.00; 30.00)	0.1995
	O	19.23 ± 5.60 (17.92; 20.54)	19.00 (15.00; 23.00)	19.11 ± 5.48 (17.28; 20.94)	20.00 (15.00; 23.00)	19.36 ± 5.81 (17.39; 21.33)	19.00 (15.00; 23.00)	0.9120
	R	18.51 ± 6.40 (17.01; 20.00)	18.00 (15.00; 22.00)	18.19 ± 6.40 (16.06; 20.32)	18.00 (14.00; 22.00)	18.83 ± 6.47 (16.64; 21.02)	17.50 (15.00; 22.50)	0.8423
AIS		29.32 ± 7.62 (27.54; 31.09)	31.00 (25.00; 35.00)	29.41 ± 7.04 (27.06; 31.75)	29.00 (26.00; 36.00)	29.22 ± 8.27 (26.42; 32.02)	32.00 (24.00; 35.00)	0.8037
SWLS		20.18 ± 4.70 (19.08; 21.27)	20.00 (17.00; 24.00)	20.05 ± 4.78 (18.46; 21.65)	20.00 (16.00; 24.00)	20.31 ± 4.68 (18.62; 21.89)	20.50 (18.00; 23.50)	0.5953
GSES		30.10 ± 4.24 (29.11; 31.08)	30.00 (28.00; 33.00)	30.22 ± 4.33 (28.77; 31.66)	30.00 (28.00; 33.00)	29.97 ± 4.20 (28.55; 31.39)	30.00 (27.50; 32.00)	0.7904
IZZ	Total	83.15 ± 11.77 (80.40; 85.90)	83.00 (76.00; 90.00)	81.51 ± 10.57 (77.99; 92.94)	83.00 (75.00; 89.00)	84.83 ± 12.82 (80.49; 89.17)	83.50 (77.00; 91.00)	0.5111
	Eating habits	21.10 ± 4.60 (20.02; 22.17)	22.00 (18.00; 24.00)	20.41 ± 4.79 (18.81; 22.00)	19.00 (18.00; 24.00)	21.81 ± 4.34 (20.34; 23.27)	22.00 (19.50; 24.50)	0.2066
	Prophylactic behaviors	21.19 ± 3.98 (20.26; 22.12)	21.00 (19.00; 24.00)	20.73 ± 3.51 (19.56; 21.90)	21.00 (19.00; 23.00)	21.67 ± 4.41 (20.17; 23.16)	21.50 (19.00; 25.00)	0.3107
	Positive mental attitude	21.42 ± 3.94 (20.51; 22.34)	22.00 (19.00; 24.00)	21.22 ± 3.61 (20.01; 22.42)	22.00 (19.00; 24.00)	21.64 ± 4.30 (20.18; 23.09)	21.50 (19.00; 24.00)	0.7860
	Health practices	19.44 ± 3.45 (18.63; 20.24)	19.00 (17.00; 22.00)	19.16 ± 3.06 (18.14; 20.18)	19.00 (17.00; 22.00)	19.72 ± 3.83 (18.43; 21.02)	19.00 (16.50; 23.50)	0.5019
LOT-R		15.84 ± 3.46 (15.03; 16.64)	16.00 (14.00; 18.00)	15.49 ± 3.07 (14.46; 16.51)	16.00 (13.00; 17.00)	16.19 ± 3.84 (14.90; 17.49)	16.50 (14.00; 18.50)	0.3722

AIS, Acceptance of Illness Scale; GSES, General Self Efficacy Scale; I, internality; IQR, interquartile range; IZZ, Health Behavior Inventory scale; LOT-R, Life Orientation Test; MHLC, Multidimensional Health Locus of Control scale; O, influence of others; R, random; SD, standard deviation; SWLS, Satisfaction with Life Scale; 95%CI, 95% confidence interval.

**Table 2:** Comparison of the results of psychological tests between subjects who completed the study.

		Total (*n* = 60)		CLA group (*n* = 31)		Control group (*n* = 29)		*p*
		Mean ± SD (95%CI)	Median (IQR)	Mean ± SD (95%CI)	Median (IQR)	Mean ± SD (95%CI)	Median (IQR)	
MHLC	I	27.20 ± 4.92 (25.93; 28.47)	27.00 (24.50; 30.00)	28.10 ± 5.26 (26.17; 30.03)	29.00 (25.00; 32.00)	26.24 ± 4.40 (24.57; 27.92)	26.00 (23.00; 30.00)	0.1210
	O	19.03 ± 5.37 (17.64; 20.42)	19.00 (15.00; 23.00)	19.32 ± 5.51 (17.30; 21.34)	20.00 (15.00; 24.00)	18.72 ± 5.30 (16.71; 20.74)	19.00 (15.00; 23.00)	0.6351
	R	18.22 ± 6.32 (16.58; 19.85)	17.50 (14.00; 25.50)	18.00 ± 6.79 (15.51; 20.49)	18.00 (14.00; 23.00)	18.45 ± 5.90 (16.20; 20.69)	17.00 (15.00; 22.00)	0.8183
AIS		29.38 ± 7.28 (27.50; 31.26)	31.00 (25.00; 35.00)	29.03 ± 7.32 (26.35; 31.72)	29.00 (25.00; 36.00)	29.76 ± 7.35 (26.96; 32.55)	32.00 (25.00; 35.00)	0.6041
SWLS		19.87 ± 4.91 (18.60; 21.14)	20.00 (16.00; 23.00)	19.90 ± 5.05 (18.05; 21.76)	20.00 (15.00; 24.00)	19.83 ± 4.85 (17.98; 21.67)	20.00 (17.00; 23.00)	0.7723
GSES		29.60 ± 4.09 (28.54; 30.66)	29.50 (27.00;32.00)	30.10 ± 4.52 (28.44; 31.75)	30.00 (27.00; 34.00)	29.07 ± 3.57 (27.71; 30.43)	29.00 (27.00; 31.00)	0.4267
IZZ	Total	83.63 ± 11.27 (80.72; 86.54)	83.50 (76.50; 90.00)	82.42 ± 10.42 (78.60; 86.24)	85.00 (76.00; 90.00)	84.93 ± 12.16 (80.31; 89.56)	83.00 (78.00; 92.00)	0.7559
	Eating habits	21.27 ± 4.11 (20.20; 22.33)	22.00 (18.00; 24.00)	20.74 v 3.92 (19.31; 22.18)	20.00 (18.00; 24.00)	21.83 ± 4.31 (20.19; 23.47)	22.00 (21.00; 24.00)	0.2948
	Prophylactic behaviors	21.62 ± 3.68 (20.67; 22.57)	21.00 (19.00; 24.00)	20.90 ± 3.66 (19.56; 22.25)	21.00 (19.00; 23.00)	22.38 ± 3.61 (21.01; 23.75)	22.00 (20.00; 25.00)	0.1907
	Positive mental attitude	21.33 ± 3.78 (20.36; 22.31)	21.50 (19.00; 24.00)	21.42 ± 3.45 (20.15; 22.69)	22.00 (19.00; 24.00)	21.24 ± 4.16 (19.66; 22.82)	21.00 (19.00; 24.00)	0.8179
	Health practices	19.42 ± 3.36 (18.55; 20.28)	19.00 (17.00; 22.00)	19.35 ± 2.89 (18.29; 20.42)	19.00 (17.00; 22.00)	19.48 ± 3.84 (18.02; 20.94)	19.00 (16.00; 22.00)	0.8407
LOT-R		15.38 ± 3.32 (14.52; 16.24)	16.00 (13.00; 17.50)	15.00 ± 3.11 (13.86; 16.14)	15.00 (12.00; 17.00)	15.79 ± 3.55 (14.44; 17.14)	16.00 (14.00; 18.00)	0.3016

AIS, Acceptance of Illness Scale; GSES, General Self Efficacy Scale; I, internality; IQR, interquartile range; IZZ, Health Behavior Inventory scale; LOT-R, Life Orientation Test; MHLC, Multidimensional Health Locus of Control scale; O, influence of others; R, random; SD, standard deviation; SWLS, Satisfaction with Life Scale; 95%CI, 95% confidence interval.

### 3.2 Comparison of the results of psychological tests between subjects who completed and not complete the study

The analysis revealed that women who dropped out from the project obtained higher scores in the GSES (*p* = 0.0490) and the LOT-R (*p* = 0.0087) scores than those who completed the study, indicating that those who did not complete the study had a greater sense of self-efficacy and were characterized by a higher level of optimism (Table 3).

**Table 3:** Comparison of the results of psychological tests between subjects who completed and not completed the study.

		Subjects who completed the study (*n* = 60)		Subjects who not completed the study (*n* = 13)		*p*
		Mean ± SD (95%CI)	Median (IQR)	Mean ± SD (95%CI)	Median (IQR)	
MHLC	I	27.20 ± 4.91 (25.93; 28.46)	27.00 (25.00; 30.00)	28.77 ± 4.08 (26.30; 31.24)	28.00 (25.00; 31.00)	0.3898
	O	19.03 ± 5.37 (17.64; 20.42)	19.00 (15.00; 23.00)	20.15 ± 6.75 (16.07; 24.23)	21.00 (16.00; 25.00)	0.5109
	R	18.21 ± 6.32 (16.58; 19.85)	17.50 (14.00; 22.50)	19.85 ± 6.81 (15.72; 23.96)	19.00 (17.00; 22.00)	0.4228
AIS		29.38 ± 7.28 (27.50; 31.26)	31.00 (25.00; 35.00)	29.00 ± 9.34 (23.35; 34.64)	32.00 (24.00; 36.00)	0.8454
SWLS		19.87 ± 4.91 (18.60; 21.13)	20.00 (16.00; 23.00)	21.61 ± 3.33 (19.60; 23.63)	20.00 (20.00; 24.00)	0.2032
GSES		29.60 ± 4.09 (28.54; 30.65)	29.50 (27.00; 32.00)	32.38 ± 4.31 (29.77; 34.99)	33.00 (29.00; 36.00)	0.0490
IZZ	Total	83.63 ± 11.27 (80.72; 86.54)	83.50 (76.50; 90.00)	80.92 ± 14.16 (72.36; 89.48)	79.00 (68.00; 90.00)	0.4190
	Eating habits	21.26 ± 4.11 (20.20; 22.33)	22.00 (18.00; 24.00)	20.31 ± 6.53 (16.35; 24.26)	19.00 (15.00; 25.00)	0.5194
	Prophylactic behaviors	21.62 ± 3.69 (20.67; 22.56)	21.00 (19.00; 24.00)	19.23 ± 4.83 (16.31; 22.15)	19.00 (17.00; 22.00)	0.0618
	Positive mental attitude	21.33 ± 3.78 (20.35; 22.31)	21.50 (19.00; 24.00)	21.84 ± 4.75 (18.97; 24.72)	22.00 (19.00; 25.00)	0.6905
	Health practices	19.42 ± 3.36 (18.54; 20.29)	19.00 (17.00; 22.00)	19.53 ± 3.99 (17.13; 21.95)	19.00 (16.00; 23.00)	0.9884
LOT-R		15.38 ± 3.32 (14.52; 16.24)	16.00 (13.00; 17.50)	17.92 ± 3.45 (15.84; 20.01)	18.00 (17.00; 19.00)	0.0087

AIS, Acceptance of Illness Scale; GSES, General Self Efficacy Scale; I, internality; IQR, interquartile range; IZZ, Health Behavior Inventory scale; LOT-R, Life Orientation Test; MHLC, Multidimensional Health Locus of Control scale; O, influence of others; R, random; SD, standard deviation; SWLS, Satisfaction with Life Scale; 95%CI, 95% confidence interval.

### 3.3 Comparison of subjects who decreased and increased body weight after the intervention period

There were no differences in the psychological test results in the total population between subjects who decreased and increased body weight after the intervention period (Table 4). Similarly, there were no differences in the results of the psychological questionnaires between participants who decreased and increased body weight, both in the CLA and control groups (Table 5).

**Table 4:** Comparison of the results of psychological tests between subjects who decreased and increased body weight.

		Body weight increase (*n* = 33)		Body weight decrease (*n* = 27)		*p*
		Mean ± SD (95%CI)	Median (IQR)	Mean ± SD (95%CI)	Median (IQR)	
MHLC	I	27.18 ± 4.93 (25.43; 28.93)	27.00 (24.00; 30.00)	27.22 ± 4.99 (25.25; 29.19)	27.00 (25.00; 30.00)	0.9347
	O	19.94 ± 5.18 (18.10; 21.78)	21.00 (15.00; 23.00)	17.93 ± 5.50 (15.75; 20.10)	17.00 (14.00; 21.00)	0.1800
	R	17.91 ± 6.36 (15.65; 20.16)	17.00 (14.00; 22.00)	18.59 ± 6.38 (16.07; 21.12)	19.00 (14.00; 23.00)	0.6233
AIS		29.55 ± 6.75 (27.15; 31.94)	31.00 (24.00; 35.00)	29.19 ± 8.01 (26.02; 32.35)	31.00 (25.00; 35.00)	0.9822
SWLS		5.15 ± 1.68 (4.56; 5.75)	5.00 (4.00; 6.00)	5.52 ± 1.74 (4.83; 6.21)	5.00 (4.00; 7.00)	0.8028
GSES		29.61 ± 3.72 (28.29; 30.92)	30.00 (28.00; 32.00)	29.59 ± 4.58 (27.78; 31.40)	29.00 (27.00; 32.00)	0.7091
IZZ	Total	84.58 ± 10.72 (80.78; 88.38)	85.00 (78.00; 90.00)	82.48 ± 12.02 (77.73; 87.24)	83.00 (75.00; 89.00)	0.6131
	Eating habits	21.18 ± 3.95 (19.78; 22.58)	21.00 (19.00; 24.00)	21.37 ± 4.38 (19.64; 23.10)	22.00 (18.00; 24.00)	0.7824
	Prophylactic behaviors	22.15 ± 3.68 (20.85; 23.45)	21.00 (19.00; 25.00)	20.96 ± 3.65 (19.52; 22.41)	21.00 (19.00; 24.00)	0.4642
	Positive mental attitude	21.85 ± 3.52 (20.60; 23.10)	22.00 (19.00; 24.00)	20.70 ± 4.06 (19.10; 22.31)	21.00 (19.00; 24.00)	0.3509
	Health practices	19.39 ± 3.45 (18.17; 20.62)	19.00 (17.00; 21.00)	10.44 ± 3.31 (18.14; 20.75)	19.00 (17.00; 22.00)	0.9463
LOT-R		15.70 ± 3.69 (14.39; 17.00)	16.00 (13.00; 18.00)	15.00 ± 2.84 (13.88; 16.12)	15.00 (13.00; 17.00)	0.3136

AIS, Acceptance of Illness Scale; GSES, General Self Efficacy Scale; I, internality; IQR, interquartile range; IZZ, Health Behavior Inventory scale; LOT-R, Life Orientation Test; MHLC, Multidimensional Health Locus of Control scale; O, influence of others; R, random; SD, standard deviation; SWLS, Satisfaction with Life Scale; 95%CI, 95% confidence interval.

**Table 5:** Comparison of the results of psychological tests between subjects in the intervention and control groups who decreased and increased body weight.

		CLA group (*n* = 31)				*p*	Control group (*n* = 29)				*p*
		Body weight increase (*n* = 14)		Body weight decrease (*n* = 17)			Body weight increase (*n* = 19)		Body weight decrease (*n* = 10)		
		Mean ± SD (95%CI)	Median (IQR)	Mean ± SD (95%CI)	Median (IQR)		Mean ± SD (95%CI)	Median (IQR)	Mean ± SD (95%CI)	Median (IQR)	
MHLC	I	29.07 ± 5.44 (25;92; 32.21)	30.00 (28.00; 32.00)	27.29 ± 5.13 (24.65; 29.93)	27.00 (25.00; 29.00)	0.1821	25.79 ± 4.13 (23.80; 27.78)	26.00 (23.00; 29.00)	27.10 ± 5.00 (23.52; 30.68)	28.00 (22.00; 30.00)	0.4750
	O	19.64 ± 5.60 (16.41; 22.87)	20.00 (15.00; 24.00)	19.06 ± 5.59 (16.18; 21.94)	20.00 (15.00; 23.00)	0.7501	20.16 ± 4.99 (17.75; 22.56)	21.00 (15.00; 23.00)	16.00 ± 5.01 (12.42; 19.58)	15.50 (12.00; 19.00)	0.0652
	R	16.57 ± 6.65 (12.73; 20.41)	17.50 (11.00; 21.00)	19.18 ± 6.67 (15.64; 22.71)	19.00 (15.00; 23.00)	0.3925	18.89 ± 6.13 (15.94; 21.85)	17.00 (15.00; 23.00)	17.60 ± 5.66 (13.55; 21.65)	19.00 (12.00; 22.00)	0.9267
AIS		28.36 ± 6.83 (24.41; 32.30)	28.00 (22.00; 35.00)	29.59 ± 7.86 (25.55; 33.63)	30.00 (28.00; 36.00)	0.4738	30.42 ± 6.74 (27.17; 33.67)	32.00 (24.00; 37.00)	28.50 ± 8.64 (22.32; 34.68)	32.00 (25.00; 35.00)	0.7128
SWLS		18.50 ± 3.99 (16.19; 20.81)	19.00 (15.00; 21.00)	21.06 ± 5.63 (18.16; 23.95)	21.00 (16.00; 24.00)	0.2095	20.05 ± 5.39 (17.45; 22.65)	21.00 (17.00; 24.00)	19.40 ± 3.84 (16.66; 22.14)	19.50 (16.00; 23.00)	0.4611
GSES		30.21 ± 3.46 (28.21; 32.22)	30.50 (28.00; 33.00)	30.00 ± 5.33 (27.25; 32.74)	29.00 (27.00; 35.00)	0.8733	29.16 ± 3.92 (27.27; 31.05)	30.00 (27.00; 31.00)	28.90 ± 3.00 (26.76 ± 31.04)	28.50 (27.00; 32.00)	0.6115
IZZ	Total	84.21 ± 6.65 (80.37; 88.06)	85.00 (79.00; 90.00)	89.94 ± 12.75 (74.38; 87.50)	83.00 (75.00; 89.00)	0.6765	84.84 ± 13.11 (78.52; 91.16)	82.00 (76.00; 95.00)	85.10 ± 10.77 (77.39; 92.81)	83.50 (79.00; 89.00)	0.9634
	Eating habits	21.07 ± 3.27 (19.18; 22.96)	19.00 (19.00; 25.00)	20.47 ± 4.46 (18.18; 22.76)	22.00 (18.00; 23.00)	0.5885	21.26 ± 4.47 (19.11; 23.42)	22.00 (17.00; 24.00)	22.90 ± 3.98 (20.05; 25.75)	22.50 (22.00; 25.00)	0.3196
	Prophylactic behaviors	22.14 ± 3.03 (20.36; 23.89)	21.00 (21.00; 23.00)	19.88 ± 3.90 (17.87; 21.89)	20.00 (16.00; 23.00)	0.1728	22.16 ± 4.17 (20.15; 24.17)	21.00 (19.00; 26.00)	22.80 ± 2.35 (21.12; 24.48)	22.00 (21.00; 24.00)	0.5044
	Positive mental attitude	22.36 ± 2.76 (20.76; 23.95)	22.50 (20.00; 25.00)	20.65 ± 3.84 (18.67; 22.61)	20.00 (19.00; 24.00)	0.2310	21.47 ± 4.02 (19.54; 23.41)	21.00 (18.00; 24.00)	20.80 ± 4.61 (17.50; 24.10)	22.00 (19.00; 23.00)	0.8900
	Health practices	18.64 ± 2.34 (17.29; 19.99)	19.00 (17.00; 19.00)	19.94 ± 3.23 (18.28; 21.60)	21.00 (18.00; 22.00)	0.1867	19.95 ± 4.05 (18.00; 21.90)	21.00 (18.00; 23.00)	18.60 ± 3.44 (16.14; 21.06)	18.00 (16.00; 19.00)	0.2477
LOT-R		15.71 ± 2.89 (14.04; 17.38)	16.00 (14.00; 17.00)	14.11 ± 3.24 (12.74; 16.08)	14.00 (12.00; 17.00)	0.2467	15.68 ± 4.26 (13.63; 17.74)	17.00 (12.00; 19.00)	16.00 ± 1.70 (14.78; 17.22)	15.50 (15.00; 17.00)	0.9083

AIS, Acceptance of Illness Scale; GSES, General Self Efficacy Scale; I, internality; IQR, interquartile range; IZZ, Health Behavior Inventory scale; LOT-R, Life Orientation Test; MHLC, Multidimensional Health Locus of Control scale; O, influence of others; R, random; SD, standard deviation; SWLS, Satisfaction with Life Scale; 95%CI, 95% confidence interval.

### 3.4 Linear regression analysis

Univariate linear regression analysis was subsequently performed to assess the relationship between age, group and results of psychological tests with body weight changes after the intervention. Table 6 shows that the SWLS results (*p* = 0.0290) were significantly associated with body weight changes. The variables from univariate analysis with *p* < 0.1 (MHLC O and SWLS), as well as age and group, were subsequently entered into multivariate linear regression (Table 7) demonstrating that the SWLS results (*p* = 0.0345) were independent negative predictors of body weight changes. Higher levels of life satisfaction were associated with more significant weight loss after the intervention.

**Table 6:** Univariate linear regression analysis for the various different variables associated with body weight changes.

		β	SE	*t*	*p*
MHLC	I	0.055	0.131	0.419	0.6766
	O	0.248	0.127	1.953	0.0557
	R	0.140	0.130	1.075	0.2870
AIS		0.178	0.129	1.380	0.1729
SWLS		−0.282	0.126	−2.240	0.0290
GSES		0.028	0.131	0.213	0.8318
IZZ	Total	0.113	0.130	0.868	0.3890
	Eating habits	−0.106	0.131	−0.813	0.4197
	Prophylactic behaviors	0.163	0.129	1.261	0.2125
	Positive mental attitude	0.197	0.129	1.529	0.1318
	Health practices	0.109	0.130	0.838	0.4054
LOT-R		0.039	0.131	0.299	0.7657
Group (CLA vs. Placebo)		−0.185	0.128	−1.447	0.1533
Age		0.110	0.129	0.847	0.4006

AIS, Acceptance of Illness Scale; GSES, General Self Efficacy Scale; I, internality; IZZ, Health Behavior Inventory scale; LOT-R, Life Orientation Test; MHLC, Multidimensional Health Locus of Control scale; O, influence of others; OR, odds ratio; R, random; SE, standard error; SWLS, Satisfaction with Life Scale.

**Table 7:** Multivariate linear regression analysis for the various different variables associated with body weight changes.

	β	SE	*t*	*p*
MHLC O	0.231	0.136	1.694	0.0959
SWLS	−0.266	0.123	−2.168	0.0345
Group (CLA vs. Placebo)	−0.202	0.123	−1.644	0.1061
Age	0.028	0.136	0.209	0.8355

MHLC, Multidimensional Health Locus of Control scale; O, influence of others, OR, odds ratio; SE, standard error; SWLS, Satisfaction with Life Scale.

## 4 Discussion

Here, we found that psychological factors like high self-efficacy and optimism levels may determine the risk of discontinuation of the intervention, while satisfaction with life may impact the effectiveness of the intervention in body weight reduction.

To obtain body weight reduction, 74 women included in this three-month randomized trial were divided into two groups receiving, respectively, capsules containing CLA or sunflower oil. However, as we reported previously (25, 26), a 12-week intervention with CLA supplementation was ineffective in reducing anthropometric indicators including body weight, BMI and waist circumference, as we observed no differences between the intervention and placebo groups. However, significant differences between groups were found in hip circumference (25) and body composition parameters (total body fat, android adipose tissue, gynoid adipose tissue, visceral adipose tissue and lean body mass to height) (26). These results were in contrast to the previous meta-analysis which reported that CLA supplementation significantly reduced body weight and BMI in participants with metabolic syndrome (34). Another meta-analysis conducted by Onakpoya et al. (20) assessed long-term (> 6 months) effects of CLA and also noticed a significant reduction in body weight and fat mass in excessive body weight subjects. However, the authors of this meta-analysis emphasized that these effects are small, therefore, the clinical relevance is uncertain. The next meta-analysis noted that supplementation with CLA, slightly but not clinically relevant, reduced body weight and fat mass, as well as increased lean body mass in overweight and obese subjects (35). Also, a recent meta-analysis demonstrated an improvement in anthropometric parameters and body composition after CLA supplementation in adults (36). However, Liang et al. (21), in their meta-analysis, demonstrated that CLA supplementation during exercise programs did not improve body weight but significantly reduced fat mass. Taking into account the inconsistency in the obtained results we hypothesized that psychological factors might affect the success of the CLA intervention and ability to complete the study.

Self-efficacy refers to the level of confidence a subject possesses in their ability to successfully engage in behaviors that enable them to accomplish specific tasks (37). This confidence can be specific to a particular domain or include a general belief about the likelihood of success in various activities (38). It was suggested that subjects with a higher level of self-efficacy are more likely to be successful during a behavioral change intervention (37). Moreover, a higher level of self-efficacy is more likely to adopt and engage in various health behaviors (39, 40). However, studies assessing self-efficacy’s role in body weight reduction provide unequivocal results (41–44). Some studies reported no association between baseline self-efficacy and weight change (41), while others found that higher baseline self-efficacy was associated with more significant weight loss (43, 44) Conversely, some researchers suggested that higher self-efficacy at baseline was associated with lower weight loss (12, 42). Self-efficacy was also related to the ability to weight loss maintenance. Mishali et al. (45) found that subjects who managed to maintain their weight reduction 2–5 years after the bariatric surgery had higher general self-efficacy than those who did not. In addition, recently, self-efficacy was found to be a strong factor of health behavior change in overweight and obese subjects (46). Our study, however, did not show an association between self-efficacy and the ability to reduce body weight after CLA supplementation in overweight and obese women. Nevertheless, self-efficacy may determine success in completing the intervention, as we observed that subjects who did not complete the study had a greater level of self-efficacy. Our findings are in contrast to previous results. Björkman et al. (13) reported that subjects with early attrition scored lower on self-efficacy compared to those patients who completed the 12-month treatment. However, this study assessed self-efficacy using other methods than in our project. Our findings can be explained by the fact that high baseline self-efficacy in the context of weight loss efforts might suggest either overconfidence or a limited understanding of the challenges associated with weight management (47).

Dispositional optimism refers to psychological factors that involve a general belief or expectation that positive outcomes, rather than negative outcomes, will prevail in one’s future (48). This trait has been associated with various effects on health behaviors, emotional wellbeing, and health outcomes (49). Dispositional optimism may also be linked to a reduced risk of some diseases and mortality rates (50). Several studies also investigated the association between optimism level and body weight, with most reporting a negative association between body weight and optimism level, especially in women (51, 52). Robert et al. (53) showed that individuals with a more optimistic outlook were less prone to being underweight or obese in comparison to those with a less optimistic disposition, whereas Fontaine and Cheskin (54) noted that the overall LOT-R and optimism subscales did not correlate with either attendance or weight reduction. Herein, we did not observe any differences in the LOT-R test results between subjects who decreased and increased body weight after the intervention period. Nevertheless, subjects who dropped out obtained higher scores in the LOT-R tests and were characterized by higher optimism levels. This is one of the first studies which compared the LOT-R test results between subjects who completed and did not complete the trial. Unfortunately, the number of subjects who dropped out of the study was relatively small compared to the group that completed the study, therefore, the apparent difference between subjects could be due to random chance. Indeed, optimistic subjects were previously considered to be more persistent in achieving their goals than less optimistic subjects (55). Moreover, some studies showed that optimism reduces dropout intentions (56) and actual college education dropout among students (57).

Health behaviors encompass all actions related to a subject’s health, which can positively or negatively affect their wellbeing (58). Engaging in pro-health behaviors contributes to the improvement of one’s overall health, whereas engaging in detrimental health behaviors can have adverse consequences on one’s wellbeing (59). Moreover, individuals who are highly motivated to improve their health may be more likely to complete the study, resulting in lower drop-out rates, whereas less motivated or interested individuals may be more inclined to drop out (60, 61). Our study, however, did not confirm this hypothesis as we showed no differences in total scores in the IZZ test and the scores obtained in each domain (eating habits, prophylactic behaviors, positive mental attitude, health practices) between subjects who completed and did not complete the study and between participants who decreased and increased body weight after the intervention period.

Locus of control refers to the belief subjects have in the amount of control they have over their lives (62). Based on the MHLC questionnaire, the locus of control can be divided into three dimensions: internal locus of control, chance or random locus of control and powerful others (external) locus of control (63). The internal locus of control seems to be more advantageous, as subjects who hold the belief that they are accountable for their health tend to exhibit healthier behaviors more frequently (64). In contrast, people with an external locus of control believe that their health is influenced by others, e.g., health professionals (65). Subjects with a random locus of control believe that health outcomes are determined by chance, luck, fate, or randomness and may be less likely to take preventative measures since they feel health outcomes are largely unpredictable or uncontrollable (63). Previous studies showed that subjects with excessive body weight are both more externally (66, 67) and more internally (68) oriented than non-obese subjects. Furthermore, a recent meta-analysis reported that an external locus of control was inversely correlated with healthy lifestyle behavior, including diet and physical activity, but was positively associated with BMI in individuals with type 2 diabetes mellitus (69). Moreover, studies that investigated the relationship between locus control and body weight reduction reported inconsistent findings. An internal locus of control was identified as a predictor of body weight decrease in several (11, 70), albeit not all, studies (13). Adolfsson et al. (11) found that body weight reduction was significantly associated with an internal locus of control among participants in a behavior modification weight loss program. In addition, Gierszewski (70) showed a negative relationship between social support and weight reduction in the case of an internal locus of control for subjects who participate in nutrition and weight reduction program. Contrary, Björkman et al. (13) found no statistically significant associations between body weight decrease after a very low-energy diet intervention and locus of control. However, women who completed the treatment had higher locus of control scores than women who dropped out. These results are partly in line with our results as we found no differences in scores obtained in the MHLC scale between subjects who successfully decreased and increased body weight. However, we also did not note differences in locus of control between participants who completed and did not complete the intervention.

Acceptance of the disease and satisfaction with life are two very important factors affecting the mental state (71). Previously, Katsaiti (72) reported that excessive body weight had a detrimental impact on life satisfaction, whereas Wang et al. (73) showed that greater adiposity was associated with lower quality of life but not life satisfaction in elderly subjects. Moreover, Urbano-Mairena et al. (74) showed that optimal body weight had a positive impact on life satisfaction in children compared to overweight and obese individuals. In addition, Górczewska and Jakubowska-Pietkiewicz (71) found that subjects with malnutrition had the lowest acceptance of the disease, while overweight women presented the lowest satisfaction with life. These factors also offer insights into the subject’s adaptation to the disease and, therefore, may determine the effectiveness of body weight reduction intervention. When subjects accept their health condition, they are more likely to engage in behaviors aimed at managing and improving their health which could include activities leading to body weight reduction (75, 76). Moreover, subjects who are more satisfied with their lives may be more motivated to care of their health, which could involve maintaining a healthy diet and regular physical activity (77). Indeed, we demonstrated that the SWLS test results but not the AIS test were independent predictors of body weight changes.

Our study has some limitations, including the small sample size, short intervention period and lack of information about the adherence levels. Moreover, to get a more homogeneous population, our study was conducted in overweight or obese women. Therefore, our results may not be generalized to other populations. Besides, psychological factors after the intervention were not evaluated to determine how the intervention affected psychological outcomes. Furthermore, we only evaluated general self-efficacy and did not determine situation-specific self-efficacy. Additionally, the number of subjects who dropped out of the study was relatively small compared to the group that completed the study. Therefore, the apparent difference between subjects who completed and did not complete the trial could be due to random chance. The use of sunflower oil as a placebo might also be considered as a study limitation as this oil contains linoleic acid, which can be partly biohydrogenated by the bacteria into CLA (78).

This is the first study that analyzed how psychological factors may determine the effectiveness of CLA supplementation in body weight reduction in overweight and obese women and the psychological factors responsible for the discontinuation of the intervention. Although we used CLA as a supplement in the study, the findings appear to be broadly applicable to other weight-loss research, as the obtained results emphasize the importance of a multidisciplinary approach in clinical studies. The strengths of the study also included strictly defined inclusion and exclusion criteria and a homogeneous population. Moreover, this well-designed randomized controlled trial was performed per CONSORT guidelines (23).

## 5 Conclusion

In conclusion, psychological factors like self-efficacy and optimism may be associated with a higher risk of withdrawal from the study, while satisfaction with life may have an impact on the effectiveness of body weight reduction. However, further larger studies are needed to confirm these findings.

## Data availability statement

The raw data supporting the conclusions of this article will be made available by the authors, without undue reservation.

## Ethics statement

The studies involving humans were approved by the Ethics Committee of the Poznan University of Medical Sciences. The studies were conducted in accordance with the local legislation and institutional requirements. The participants provided their written informed consent to participate in this study.

## Author contributions

MJ: Data curation, Formal analysis, Writing – original draft. JP: Data curation, Writing – review & editing. AB-P: Investigation, Methodology, Writing – review & editing. EM: Conceptualization, Investigation, Methodology, Writing – review & editing. AL: Methodology, Writing – review & editing. KJ-P: Writing – review & editing. JC-P: Writing – review & editing. PB: Writing – review & editing. JW: Conceptualization, Data curation, Funding acquisition, Methodology, Project administration, Writing – original draft.
